# *In vitro* antimalarial susceptibility profile of *Plasmodium falciparum* isolates in the BEI Resources repository

**DOI:** 10.1128/aac.01189-23

**Published:** 2024-09-13

**Authors:** Amel O. A. Ahmed, Standwell C. Nkhoma, Sharmeen Zaman, Sujatha Rashid, Rebecca Bradford, Timothy T. Stedman, Robert E. Molestina

**Affiliations:** 1 BEI Resources, Manassas, Virginia, USA; 2 ATCC, Manassas, Virginia, USA; The Children's Hospital of Philadelphia, Philadelphia, Pennsylvania, USA

**Keywords:** malaria, drug susceptibility, resistance, standards, BEI Resources, *P. falciparum*

## Abstract

BEI Resources, a National Institute of Allergy and Infectious Diseases-funded program managed by the American Type Culture Collection, serves researchers worldwide through the provision of a centralized repository for the acquisition, production, characterization, preservation, storage, and distribution of standardized biological resources targeting National Institutes of Health priority pathogens including bacteria, viruses, pathogenic fungi, and parasitic protozoa. These reference materials are critical for the development of diagnostics, vaccines, and therapeutics and are available to qualified registered investigators and institutions worldwide. Bioresources within BEI include well-characterized malaria isolates as part of the Malaria Research and Reference Reagent Resource Center (MR4). These isolates are critical for screening antimalarial compounds, conducting drug resistance studies, and for resistance surveillance and management. In our efforts to enhance the characterization of MR4 *P. falciparum* isolates, we measured antimalarial susceptibility of >100 isolates against a panel of standard antimalarial compounds. Our results provide valuable information to assist current and prospective users of the BEI Resources repository in making data-driven requests of isolates to meet their research needs.

## INTRODUCTION

Malaria, caused by the protozoan parasite *Plasmodium falciparum*, is one of the most debilitating mosquito-borne diseases and continues to pose a major health and socio-economic burden in endemic countries. The World Health Organization (1) estimates that in 2021, there were 247 million malaria cases globally and 593,000 deaths in Africa, where >90% of mortality occurs (1). BEI Resources (https://www.beiresources.org), a program managed by the American Type Culture Collection since 2003 with funding and support from the National Institute of Allergy and Infectious Diseases, covers a diverse array of priority pathogens including emerging infectious disease agents, non-pathogenic microbes, and other microbiological materials of relevance to the infectious disease research community, including *P. falciparum* strains and derived reagents. The Malaria Research and Reference Reagent Resource Center (MR4) was integrated into the BEI Resources Program in 2010. In addition to providing bioresources for vector research [Vector Resources (beiresources.org)], rodent malaria parasites, mosquito cell lines, transfection vectors, and antibodies, MR4 supplies drug-susceptible and resistant *P. falciparum* strains. These resources are urgently needed to address the growing problem of antimalarial resistance, which is a major impediment to malaria control and elimination efforts. Resistance has now emerged to all antimalarial drugs in clinical use including artemisinin (ART) (2–5), the key component drug of artemisinin-based combination therapies, used worldwide (6, 7). Ensuring the availability of a carefully selected set of both drug-susceptible and resistant *P. falciparum* strains is critical for screening candidate antimalarials (8–10), for conducting basic research on the mechanisms of drug action and resistance (10–13), and for resistance surveillance and management (14, 15). Concerted efforts are made to acquire, produce, characterize, preserve, and distribute both sensitive and drug-resistant isolates, which have been carefully selected to meet the needs of the malaria research and control community. As part of our commitment to providing well-characterized reference malaria strains, in 2012, we implemented *in vitro* antimalarial susceptibility testing on isolates acquired from different malaria-endemic countries and laboratories worldwide; and deposited into the MR4 within the BEI Resources repository. Standardized SYBR Green I assays (16, 17) were used to determine the susceptibility of 109 isolates against a standard panel of drugs including chloroquine (CQ), quinine (QN), pyrimethamine (PYR), sulfadoxine (SDX), and cycloguanil (CYC). These assays measure the half-maximal inhibitory concentration (IC_50_), the concentration of the drug that inhibits parasite growth by 50%. For a subset of isolates (*n* = 26), additional susceptibility testing was performed against a secondary panel of drugs including lumefantrine (LM) and halofantrine (HF). Because standard IC_50_ assays poorly capture parasite susceptibility to artemisinin drugs and piperaquine (18, 19), ring-stage survival assays and piperaquine-survival assays were also performed on a subset of isolates, which were primarily deposited into the BEI Resources repository as showing susceptibility or resistance to each of these drugs. Our data sets identify parasites with unique susceptibility profiles that any registered investigator or institution can request from BEI Resources.

## MATERIALS AND METHODS

### Antimalarial test compounds

CQ, ART, QN, LM, HF, and SDX were obtained from Sigma-Aldrich; CYC was obtained from Toronto Research Chemicals; and PYR was obtained from MP Biomedicals. Dihydroartemisinin (DHA) and piperaquine (PPQ) were obtained from the WorldWide Antimalarial Resistance Network (https://www.wwarn.org/). All other chemicals used were standard commercial products of analytical grade. Drug stock solutions were prepared at 10 mM in distilled water (CQ), 70% ethanol (ART, QN, and PYR), or dimethyl sulfoxide (DMSO) (CYC, LM, and HF) except SDX stock prepared at 100 mM in DMSO. DHA stock solution was prepared at 700 µM and PPQ at 200 µM in DMSO and 0.5% lactic acid, respectively.

### Selection and *in vitro* culture of *Plasmodium falciparum* strains

*Plasmodium falciparum* isolates (*n* = 109) from BEI Resources were used in this study (Table S1). The strains originated from diverse geographical regions and epidemiological settings and included genome-edited parasites and clones from multiple investigators and laboratories. Parasite isolates were propagated using standard culture protocols as described previously (17, 20). Briefly, parasites were grown in leukocyte-depleted human type O + erythrocytes (Grifols Bio Supplies Inc., formerly the Interstate Blood Bank Inc.) in RPMI 1640 media (Gibco, 21870–084) supplemented with 0.18% glucose (Sigma, G7021), 0.18-mM hypoxanthine (Sigma, H9636), 1.77-mM L-glutamine (Gibco, 25030–149), 22-mM HEPES buffer (Gibco, 15630–080), 0.21% sodium bicarbonate (Gibco, 21870–084), 4-µg/mL gentamicin (Gibco, 15750–060), and 10% human type A + serum (Grifols Bio Supplies Inc.) under standard *in vitro P. falciparum* culture conditions (3). *P. falciparum* asexual blood-stage parasites were propagated at 37°C in complete culture media at 2%–4% hematocrit in a hypoxic environment (5% CO_2_, 5% O_2_, and 90% N_2_) in T25 flasks.

### Standard *in vitro* antimalarial susceptibility testing

**Fig 1:**
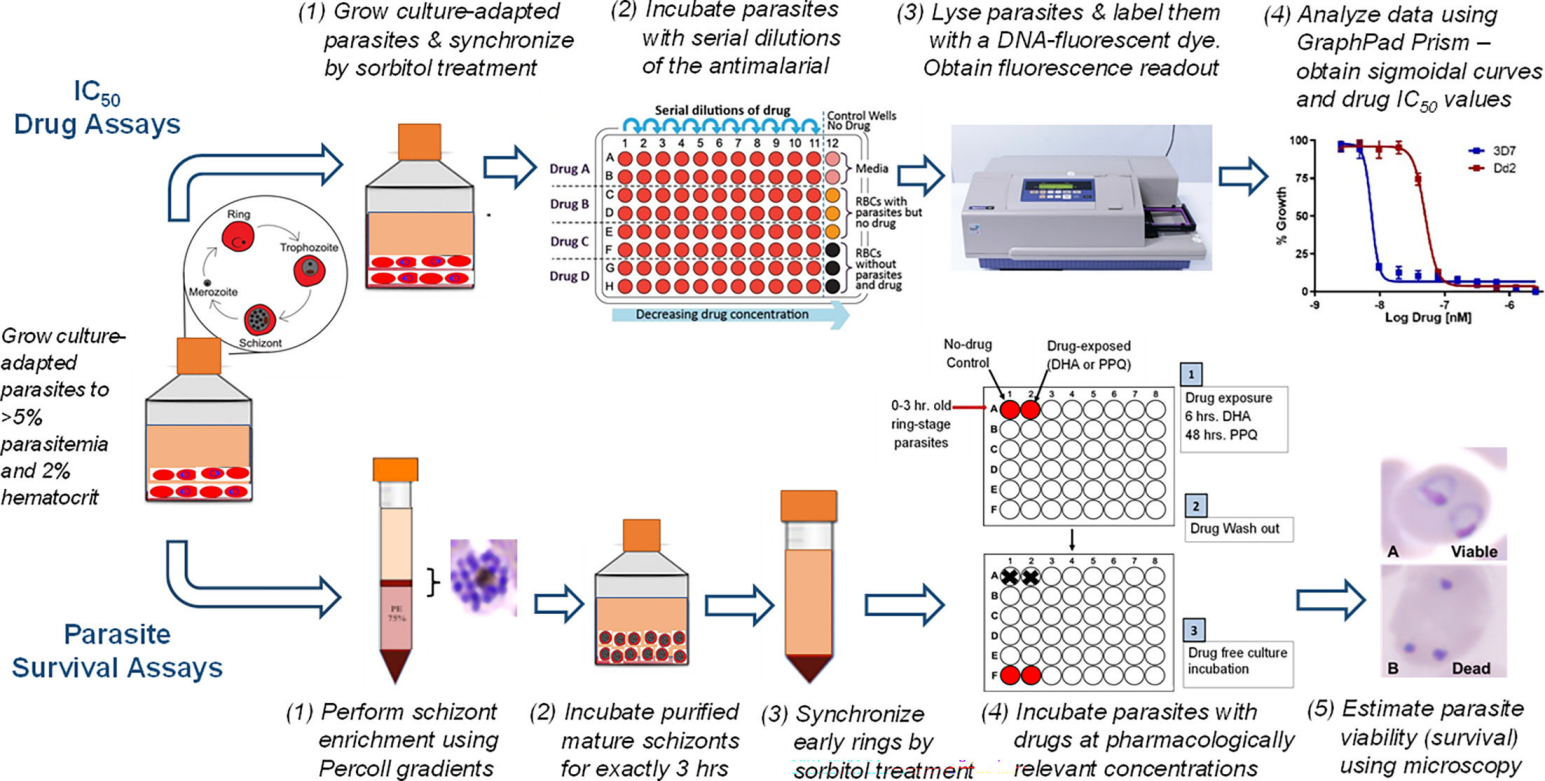
Technical approach summary. Schematic representation of antimalarial susceptibility testing for malaria isolates using standard IC_50_ drug assays (top panel, steps 1–4) and parasite survival assays (bottom panel, steps 1–5). Drug concentrations were converted to their natural logarithm to plot the sigmoidal curves and estimate the IC_50_ of each compound.

The IC_50_ of each isolate for standard antimalarial compounds CQ, ART, QN, CYC, PYR, and SDX was measured using the standardized fluorescent SYBR Green I assay as previously described (16, 17, 21). A subset of isolates (*n* = 26) was also tested against a secondary drug panel including LM and HF. At least three independent drug assays were run in duplicates at 0.5% parasitemia (≥70% ring stages) and 1.5% hematocrit in 96-well plates. Briefly, working stocks of antimalarial compounds were prepared in the drug master plate and linearly transferred to the assay plate. Final concentrations for CQ, QN, and CYC in the drug assay plate ranged from 2.44 to 2,500 nM and were 0.24–250.0 nM, 0.12–125.0 nM, 0.31–625.0 nM, 9.77–100,000.0 nM, and 12.2–1,000,000.0 nM for ART, HF, LM, PYR, and SDX, respectively. An inoculum containing parasites, blood, and culture media was added to drug assay plates, placed in a modular incubation chamber, gassed with 5% O_2_, 5% CO_2_, and 90% N_2_, and maintained at 37°C for 72 h. Each drug assay run included culture-adapted *P. falciparum* parasite lines 3D7 (MRA-102) and Dd2 (MRA-150) as reference standards. Following parasite incubation, erythrocytes were initially lysed by a freeze-thaw step, transferred to a fresh 96-well detection plate, and then labeled with a fluorophore stain, SYBR Green I (Molecular Probes, USA) at 4× dilution in 1× lysis buffer (10-mM Tris, 2.5-mM EDTA, 0.004% saponin, and 0.04% Triton X). The detection plate was incubated in the dark for 1 h, and fluorescence was measured at excitation and emission wavelengths of 490 and 540 nm, respectively, using the SpectraMax plate reader (Molecular Devices, USA) (see Fig. 1). Dose-response curves and IC_50_ values were determined from log-transformed drug concentrations and fluorescence reads at each serial drug dilution using GraphPad Prism software (GraphPad, USA).

### *In vitro* antimalarial susceptibility testing using parasite survival assays

The parasite survival rate (% S) of a subset of parasite isolates after DHA or PPQ exposure was measured using parasite survival assays, as previously described (18, 19), because standard IC_50_ assays fail to accurately capture parasite susceptibility to these compounds. Briefly, parasites were exposed to a pharmacologically relevant drug dose (700-nM DHA for 6 h or 200-nM PPQ for 48 h) in 48-well plates in a modular incubation chamber under standard culture conditions and gassed with 5% O_2_, 5% CO_2_, and 90% N_2_. The drug was then removed, and parasites were allowed to recover and proliferate in normal growth media for 66 h in ring-stage survival assay (RSA) for DHA or 24 h in piperaquine survival assay (PSA) for PPQ. Percent parasite survival was determined by examining and counting viable parasites in drug-treated versus untreated wells using light microscopy (see Fig. 1). Parasites showing a survival rate of ≥10% were deemed drug resistant based on previously defined thresholds (18, 19, 22).

## RESULTS AND DISCUSSION

**Fig 2:**
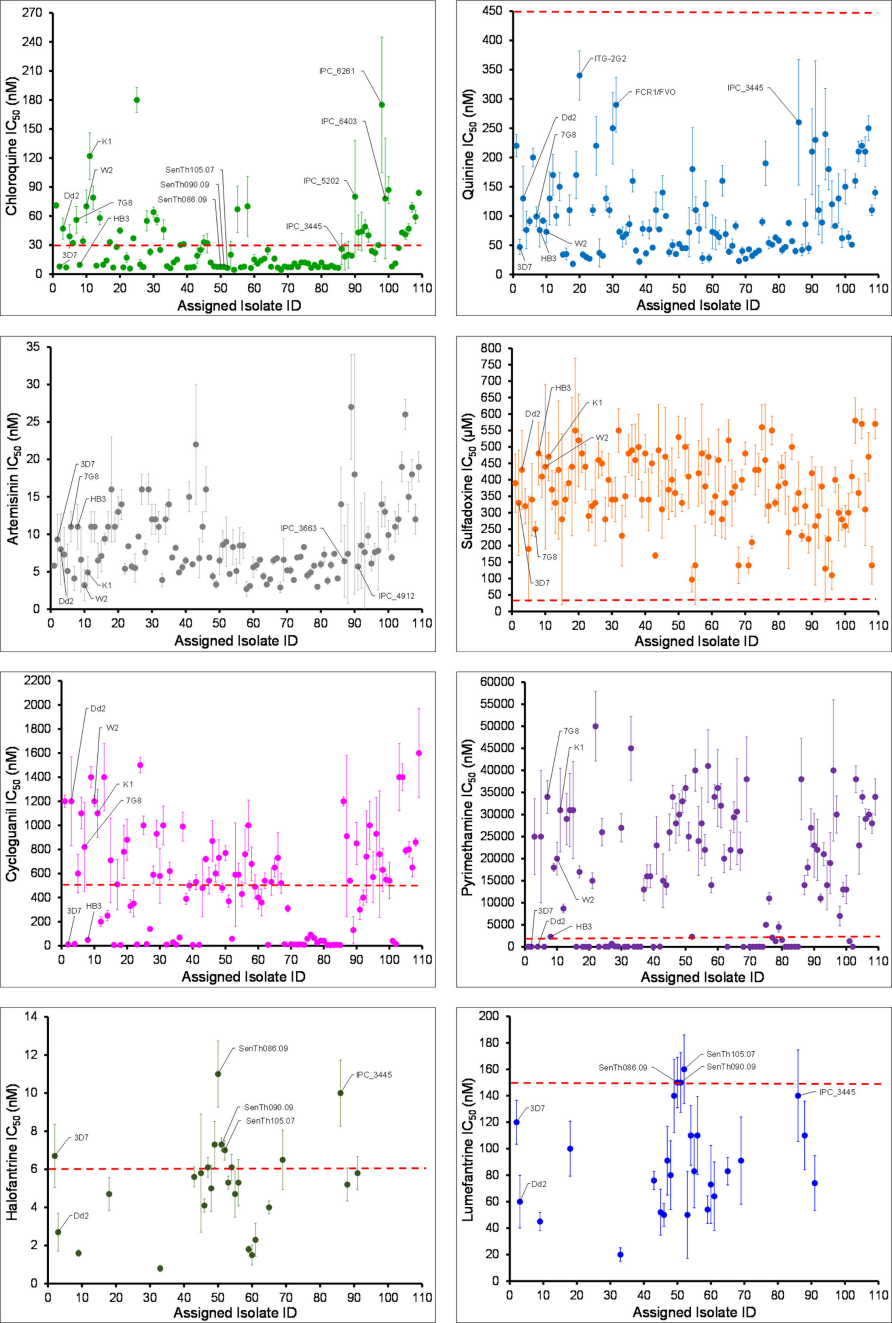
Absolute drug susceptibility of BEI *P. falciparum* isolates. Each dot on the plot represents an IC_50_ for a single isolate. The dotted red line represents the previously suggested threshold for categorizing parasites as either drug-sensitive (below the line) or resistant (above the line). Previously suggested IC_50_ thresholds for *in vitro* CQ resistance are 30–100 nM for moderate resistance (17) and >100 nM for high-grade resistance (23–25). Thresholds for QN, PYR, SDX, CYC, HF, and LM resistance are >450 nM (23–25), 2,000 nM (26), 32 µM (27), 500 nM (26), 6 nM (28, 29), and 150 nM (24), respectively. The names of common reference isolates and other isolates discussed in the text are shown on each dot of the plot at the assigned isolate ID indicated in Table S2.

Standard IC_50_ assays reveal significant variation in drug susceptibility among BEI Resources *P. falciparum* isolates, with some isolates exhibiting sensitivity and others showing resistance to a variety of antimalarial compounds tested (Fig. 2; Table S2). This extensive variation in drug response was observed across all the drugs tested except quinine, which was potent against all the isolates, and sulfadoxine, which was ineffective against all the isolates analyzed (Fig. 2). The drug susceptibility status of each isolate matched the drug response phenotype indicated by the isolate’s depositor. We observed that absolute IC_50_ values vary significantly even within the two defined categories of susceptibility and resistance (Fig. 2; Table S2), suggesting a multigenic inheritance of drug resistance.

**Fig 3:**
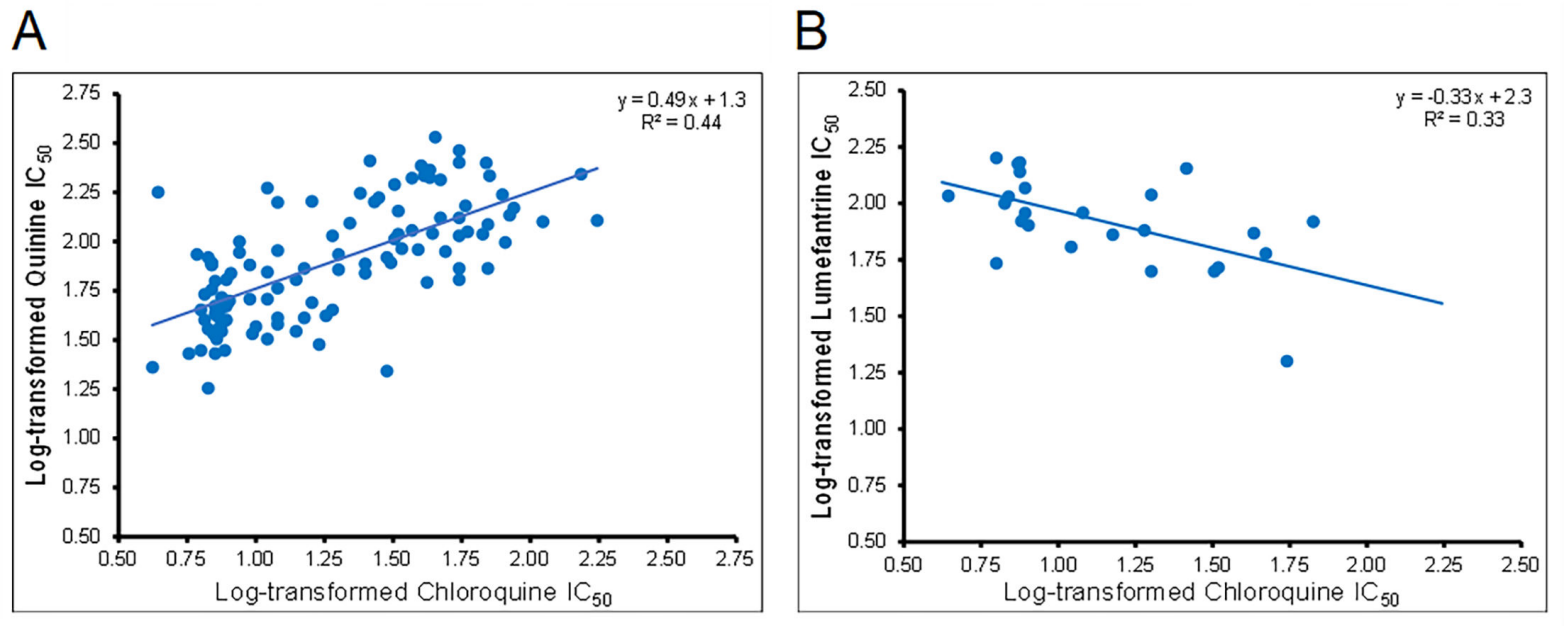
Correlation between *in vitro* activities of antimalarial drugs. Scatter plots of IC_50_ data recapitulate previously defined activity relationships between drugs. This includes the positive correlation between the potency of chloroquine (CQ) and quinine (A), and the inverse relationship between the *in vitro* activities of CQ and lumefantrine (B).

Variability in drug response is a key feature of this set of *P. falciparum* isolates and could be exploited by pre-clinical drug development programs to rationally design antimalarial compounds that limit or bypass resistance mechanisms (30), ensuring a long therapeutic lifespan of drugs emerging from the developmental pipeline. Variation in drug susceptibility also provides a unique opportunity for investigators to use these isolates as critical standards for resistance surveillance and management (14), and as controls in drug resistance studies (31). Unveiling this susceptibility data set is meant to equip current and prospective users of the BEI Resources reagents with key information to make data-driven requests to meet their research needs. The *in vitro* susceptibility data presented herein were determined from independent IC_50_ assays performed on different occasions when each isolate was replenished for distribution. Standard conditions of *in vitro* parasite culture and assessment of drug susceptibility (17, 21) were maintained for all isolates, including the different distribution batches (lots) for each isolate. This practice ensures that the data obtained are of high quality and reproducible across independent assays. Except for a few multiclonal isolates that show variable susceptibility when assayed on independent occasions [IPC_3445 (MRA-1236), IPC_5202 (MRA-1240), and IPC_6403 (MRA-1285); highlighted in Fig. 2; Table S2] (17), there was no significant variation in drug response between independent IC_50_ assays for the different distribution batches of the same isolate (one-way analysis of variance, *P* > 0.05). We observed a positive correlation between the *in vitro* activities of CQ and QN and an inverse relationship between the potency of CQ and LM (Fig. 3). These findings are consistent with cross-resistance patterns observed between these drugs in both field and laboratory-adapted isolates of *P. falciparum* (32–37). Three Senegalese isolates, SenTh086.09 (MRA-1189), SenTh090.09 (MRA-1190), and SenTh105.07 (MRA-1191), show elevated LM IC_50_ values (150 ± 19 nM, 150 ± 23 nM, and 160 ± 26 nM) but lower CQ IC_50_ values of <30 nM (7.5 ± 0.7 nM, 7.4 ± 0.5 nM, and 6.3 ± 0.3 nM), respectively (highlighted in Fig. 2; Table S2). This finding reinforces the previously known inverse relationship between CQ and LM *in vitro* activities (35, 36). A combination of LM and Artemether (CoArtem) is currently the first-line treatment for uncomplicated malaria in most malaria-endemic countries, particularly in Africa. The inverse correlation between CQ and LM IC_50_ values raises the intriguing possibility that extensive CoArtem use might drive an increase in LM resistance (38, 39) while simultaneously resulting in the loss of CQ resistance and potentiating CQ activity (40, 41). Such a scenario could lead to the restoration of CQ efficacy as was observed in Malawi (42). This would present a real prospect of rotating antimalarial drugs as a strategy for combating resistance and preserving the clinical lifespan of antimalarial drugs (39, 43). While the preceding discussion has focused on naturally occurring and gene-edited parasites, BEI Resources also carries some drug-selected parasites. For example, SB1-A6 (MRA-1002), the parasite line selected for atovaquone (ATQ) resistance from strain D6 (MRA-285), is >7,000-fold less susceptible to ATQ compared to its progenitor (44) and to 3D7 (our data, 1.2 nM versus 9.1 µM) and harbors resistance-associated mutations in the *cytochrome b* gene. Similarly, strain AM1 (MRA-1257), selected for fosmidomycin (FSM) resistance through application of drug pressure (45), shows an elevated FSM IC_50_ compared to the progenitor 3D7 strain (5.8 nM versus 1.1 µM). These lines could be useful controls for *in vitro* screening of compounds targeting critical functions of the apicoplast and mitochondria in malaria parasites.

**Fig 4:**
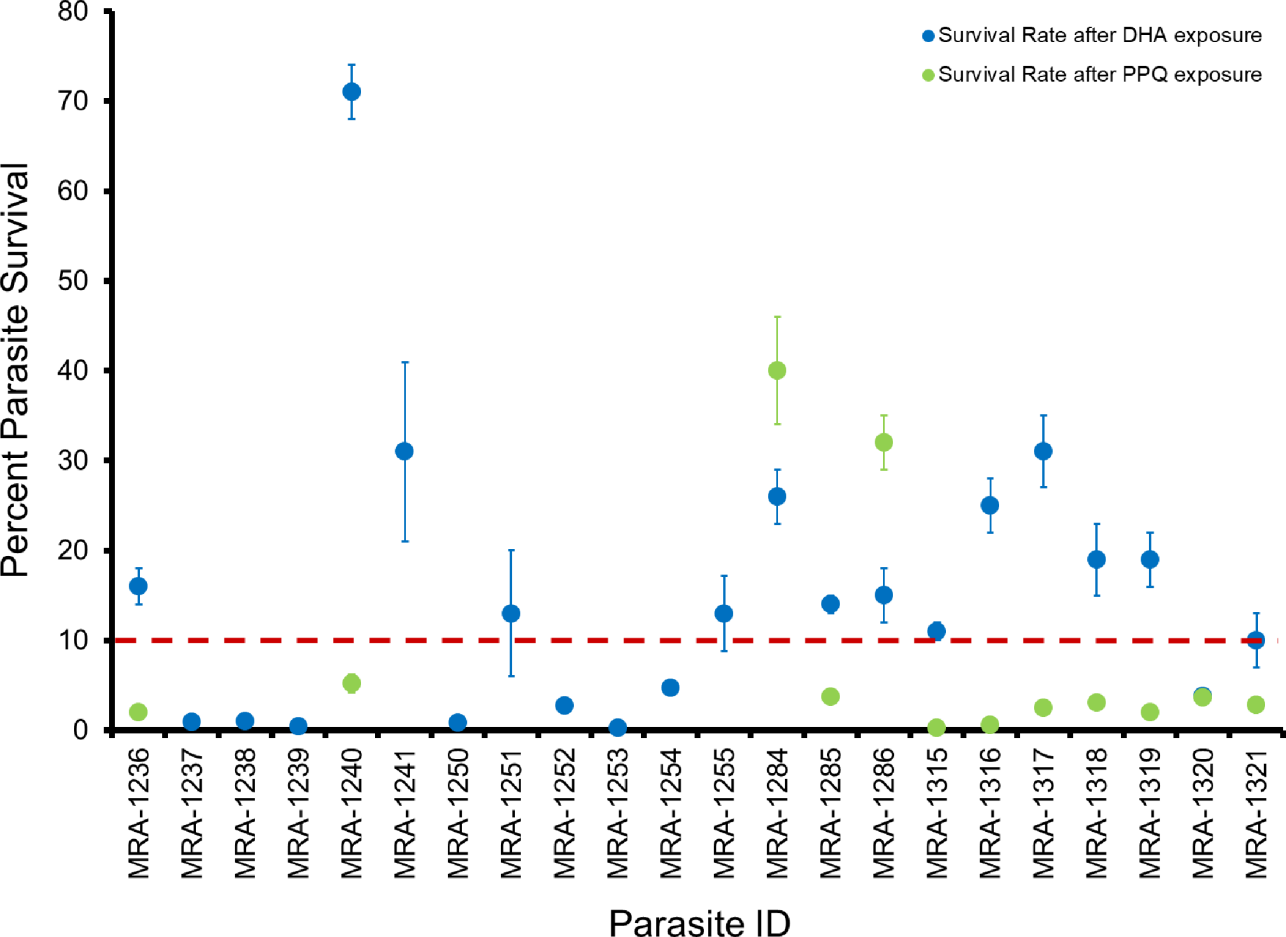
Parasite survival rates after exposure to pharmacologically relevant DHA or PPQ concentrations. Blue and green dots show data for DHA and PPQ, respectively. The dotted red line is a previously defined threshold for categorizing parasites as either drug susceptible (below the line) or drug resistant (above the line). Both susceptible and resistant isolates are adequately represented in the BEI Resources MR4 repository.

Our data show that standard IC_50_ assays poorly capture artemisinin resistance as absolute IC_50_ values for artemisinin-resistant parasites overlap those of sensitive ones, consistent with observations from previous studies (2, 18). This phenomenon is exemplified by the artemisinin-resistant parasite IPC_4912 (MRA-1241) and the artemisinin-sensitive parasite IPC_3663 (MRA-1237), which have similar IC_50_ values (5.7 ± 3.1 nM and 6.4 ± 4.8 nM, *P* > 0.05) but differ in parasite survival rates (0.9 ± 0.6 and 31 ± 10, *P* < 0.05), respectively (Fig. 2; Tables S2 and S3). Parasite survival assays, which are more informative than traditional IC_50_ assays at assessing ART and PPQ susceptibility (18, 19), revealed that 59% (*n* = 13) of the 22 isolates tested for ART susceptibility have a parasite survival rate of ≥10%, indicative of resistance. Two isolates, IPC_6261 (MRA-1284) and IPC_6293 (MRA-1286), that were primarily deposited into the BEI Resources Program based on their PPQ susceptibility show a parasite survival rate of ≥10% and are deemed PPQ resistant, while the third isolate, IPC_6403 (MRA-1285), is PPQ sensitive, with a survival rate of <10% (Fig. 4; Table S3). The two PPQ-resistant isolates also carry multiple copies of *plasmepsin II* and the H97Y *pfcrt* mutation implicated in PPQ resistance (46), while the PPQ-sensitive isolate, IPC_6403, has a single copy of *plasmepsin II* and the wild-type amino acid histidine at *pfcrt* codon 97 (Table S3). Isolates IPC_6261 and IPC_6293 also exhibit ART resistance (Fig. 4; Table S3) and harbor the artemisinin resistance-conferring C580Y mutation, raising concerns about the therapeutic lifespan of DHA-PPQ combination therapy widely used in Southeast Asia. While most ART-resistant parasites within BEI Resources have either the K13-C580Y or the K13-R539T mutation, the isolate IPC_4912 carries the K13-I543T mutation, which also confers ART resistance but is rarely seen in population surveys from the Greater Mekong Region (47, 48). These parasite strains can serve as critical controls for ART and PPQ resistance surveillance and drug resistance studies.

While the preceding discussion has highlighted the uniqueness of drug susceptibility profiles of BEI Resources isolates that could be harnessed for specific research applications, there are other advantages to using these isolates. First, the isolates undergo minimal passage to preserve their genotypic and phenotypic integrity. Producing each distribution lot from a seed vial and minimizing time in culture reduces selection pressures that could lead to genetic and/or phenotypic changes (49–51). Second, all isolates go through the same rigorous authentication (characterization) steps, including assessment of parasite viability, confirmation of the phenotype indicated by the depositor, and sequencing to ascertain genetic identity. Third, every distribution batch is tested for purity by screening for microbial and fungal contamination using a comprehensive panel of bacterial and fungal media and by molecular screening for mycoplasma contamination. All microbial contamination tests for malaria isolates reported here were negative. Traceability is another key advantage of using malaria isolates from BEI Resources. Information about the isolate such as its original name, depositor, BEI-assigned name and number, lot number, and other key attributes, including its drug susceptibility profile and sequence data, are provided in the certificate of analysis. In addition, an efficient system exists to address customer queries and provide technical support for the items ordered.

In summary, BEI Resources provides malaria isolates with unique and well-characterized drug susceptibility profiles. These isolates can be requested from BEI Resources (https://www.beiresources.org) at no additional cost to registered investigators and institutions.

## SUPPLEMENTAL MATERIAL

Supplemental material aac.01189-23-s0001.xlsx Tables S1 to S3.

ASM does not own the copyrights to Supplemental Material that may be linked to, or accessed through, an article. The authors have granted ASM a non-exclusive, world-wide license to publish the Supplemental Material files. Please contact the corresponding author directly for reuse.
